# Maspin overexpression modulates tumor cell apoptosis through the regulation of Bcl-2 family proteins

**DOI:** 10.1186/1471-2407-5-50

**Published:** 2005-05-20

**Authors:** Weiguo Zhang, Heidi Y Shi, Ming Zhang

**Affiliations:** 1 Baylor College of Medicine, Department of Molecular & Cellular Biology, Houston, TX 77030, USA

## Abstract

**Background:**

Maspin is a member of serpin family with tumor suppressing activity. Recent studies of maspin in animal models strongly support maspin's role as an inhibitor against the growth of primary tumor sand the process of metastasis. However, the molecular mechanism underlying this inhibition has not been fully elucidated. In this report, we analyze the effect of maspin on tumor cell apoptosis under several stress conditions.

**Methods:**

Stable clones overexpressing maspin are established in the mouse mammary tumor TM40D cells. They are treated with staurosporine, TNF-alpha, and serum starvation. The rates of cell apoptosis are analyzed by TUNEL assay. Inhibitors against caspase 8 and 9 are used in the apoptosis assay. Western blot analysis and ribonuclease protection assay (RPA) are performed to examine the expression of Bcl2 family genes.

**Results:**

We report that maspin expressing tumor cells have increased rate of apoptosis when they are treated with staurosporine and serum starvation. The effect is not through extracellular maspin. Maspin-mediated apoptosis is partially blocked by caspase 8 and 9 inhibitors, and is accompanied by changes in the Bcl-2 family proteins. Maspin-expressing tumor cells have a reduced level of anti-apoptotic protein Bcl-2, and an increased level of pro-apoptotic protein Bax. The regulation is not controlled at the transcriptional level but is through selective control of Bcl-2 and Bax protein stability.

**Conclusion:**

Maspin overexpression modulates tumor cell apoptosis through the regulation of Bcl2 family proteins. Such change results in an increased release of cytochrome c from mitochondria, thus the increased apoptosis in maspin-expressing cells. This evidence strongly suggests that the induction of apoptosis in maspin-overexpressing cells represents a major mechanism by which maspin inhibits breast tumor progression.

## Background

Maspin is a member of serpin family with unique tumor suppressing activity. Initially identified from normal mammary epithelial cells, maspin gene is not mutated nor deleted but is rather transcriptionally down-regulated or silenced by epigenetic changes in breast cancer [1-5]. Maspin protein made from E.*coli*, yeast, and insect inhibits breast tumor cell migration and invasion [6]. In animal models, maspin has been found to inhibit angiogenesis in rat cornea and xenograft models [7], and to inhibit mammary tumor progression and metastasis in bitransgenic mice [8]. In human breast tissue, maspin is expressed in both luminal and myoepithelial cells, and it has been suggested that the maspin-expressing myoepithelial cells form a defensive barrier for the progression from ductal carcinoma in situ to more invasive carcinoma [9]. Despite these findings, the molecular mechanism underlying maspin's inhibition is not well characterized. Previously, our laboratory showed that overexpression of maspin in normal mammary gland inhibited alveolar development during pregnancy through the induction of mammary cell apoptosis [10]. We also demonstrated that in mammary tumors isolated from WAP-SV40 TAg and WAP-maspin bitransgenic mice there was a strong correlation between maspin overexpression and increased apoptosis [8], suggesting that maspin might induce tumor cell apoptosis *in vivo*. Jiang et al also showed that maspin could sensitize MDA-435 mammary tumor cells to apoptosis induction with a chemical reagent [11]. Recently, we have established a new mammary tumor implantation model to further elucidate the mechanism of maspin-mediated tumor suppression [12]. We demonstrated that retrovirus infection of TM40D mammary tumor cells with maspin significantly blocked tumor growth and metastasis. Using the TM40D mammary tumor cells, we showed that maspin induced tumor cell apoptosis through translocation to mitochondria [13]. Here, we show that maspin-overexpressing tumor cells display a high rate of apoptosis when cells are treated with a chemical reagent staurosporine or under serum starvation. We have shown that the death signal does not involve the secreted maspin on cell surface but rather is mediated through the intracellular function of maspin. An intrinsic death signal pathway is induced which alters the protein level of Bcl-2 family members in maspin-expressing tumor cells.

## Methods

### Cell line and cell culture

Murine mammary tumor cell line TM40D cells were infected with retrovirus vector for establishing stable cell lines as described by Shi et al. [12]. Briefly, human maspin cDNA was cloned into pS2-GFP, a retroviral vector that was derived from the pS2 family of retroviral vectors. The plasmid constructs, pS2-maspin and pS2-blank vector were transfected into 293T package cells to produce infective viral particles. The viral supernatants were then allowed to infect TM40D cells in the presence of Polybrene. The transfected cells were then selected in the presence of 100 μg/ml of zeocin (Invitrogen Co., CA). Cells were seeded by limiting dilution in 96-well plates. Single clones of stably transfected cells were transferred to individual wells of 24-well plates and cultured in medium containing 100 μg/ml Zeocin. Individual clones were confirmed for the presence of human maspin cDNA by RT-PCR, immunobloting with maspin polyclonal antibody and immunofluorescence staining. Two maspin expression clones were named as TM40D-Mp (16) and TM40D-Mp (18), respectively. One TM40D cell line infected by pS2-vector was used as a negative control. All tumor subclones were maintained at 37°C in a humidified 95% O2-5% CO2 atmosphere in DMEM supplemented with 10% FBS and L-glutamine.

### Maspin immunostaining

For intracellular maspin immunostaining, cells grown on chamber slides were fixed in 4% paraformaldhyde solution for 1 hr and were permeabilized with 0.5% NP-40 in PBS for 30 min. The slides were blocked with 10% normal horse serum for 1 hour before they were treated with the primary antibody Abs4A at a dilution of 1:200. The secondary antibody (Texas red conjugated goat anti-rabbit antibody, Santa Cruz, CA) was used at a dilution of 1:1000 at room temperature for 1 hour. For cell surface staining, cells grown on slides were washed with PBS without Mg++ and Ca++ and blocked with 2% BSA for 1 hr at 4°C. First antibody was used at a dilution of 1:200. The secondary antibody (Texas red conjugated goat anti-rabbit antibody, Santa Cruz, CA) was used at a dilution of 1:500 at 4°C for 30 min. Slides were mounted and viewed under a Leica fluorescence microscope.

### Apoptosis induction

TM40D cells were plated on coverslips or 10 cm plates and cultured to 80% confluence. They were treated by one of the following procedures: 1) Serum starvation: TM40D cell lines were cultured under serum free condition for 24–72 hours in D-MEM medium with 0.1% BSA; 2) TNF-alpha treatment (modified according to Kulik's method) [14]. Briefly, the cells were incubated in serum-free DMEM medium for 12 hours, and then were treated with 100 ng/ml TNF-alpha (Sigma) for another 7 hours; 3) Staurosporine treatment: 1 μM staurosporine was added to the D-MEM medium with 2.5% FBS for another 4 hours. In conjunction with the above treatments, either the caspase-8 inhibitor II (Z-IETD-FMK) or caspase-9 inhibitor II (LEHD-CHO) were added into the media at a concentration of 20 nM.

### TUNEL assay

After the various treatments, the cells cultured on coverslips were washed with ice-cold PBS and fixed in 4% paraformaldehyde for 1 hour at 4°C. The TUNEL (Terminal deoxynucleotidyl transferase-mediated dUTP nick end labeling) assay was performed according to the manufacturer's specifications (Roche). Briefly, cell samples were fixed with freshly prepared 4% paraformaldehyde solution for 1 hour. Cells in the coverslips were rinsed with PBS twice and permeabilized with 0.1% Triton X-100 in 0.1% sodium citrate for 2 min on ice. Following the triton treatment, 50 μl TUNEL reaction mixture was added to each sample for 30 min at 37C. The slides were then rinsed 3 times with PBS and counterstained with DAPI. The mounted slides were analyzed using fluorescence microscopy. The quantitation of apoptosis was done by counting the number of apoptotic positive cells in four randomly selected fields with a 20 X objective. For antibody-blocking assay, TM40D-Mp (18) cells were cultured at 80% confluence. Cell apoptosis was induced using staurosporine (1 μM in DMEM) and the anti-maspin antibody (AbS4A) was added to the medium at a concentration of 1:200 or 1:400. Normal fetal bovine serum FBS (1:25) and rabbit IgG (1:200) were used as negative controls. After 7-hour incubation, cells were fixed and used for TUNEL assay as described above.

### Cytosolic protein extraction and cytochrome c immunoblot

Cytochrome c immunoblot was carried out using a modified protocol of Kluck et al [15]. Cultured cells were starved in serum-free DMEM medium for 48 hours and then collected with lysis buffer (1 mM CaCl2, 1 mM MgCl2, 1% NP-40, 1 μg/ml leupeptin, 1 μg/ml aprotinin, 1 μM PMSF, and 100 μM NaVO4). After the lysis mixtures were incubated on ice for 20 mins, they were centrifuged at 14,000 rpm for 15 min. The supernatants were collected and total cytosolic proteins were quantitated by a Bradford spectrometer. About 80 μg protein was loaded in a 10% SDS-PAGE gel and transferred to polyvinylidene difluoride (PVDF) membrane (Bio-rad Laboratories, Richmond, CA) at a constant current of 250 mA for 2 hours. The membrane was incubated with the primary cytochrome c polyclonal antibody (H-104, Santa Cruz) overnight at a dilution of 1:500. Horseradish peroxidase-conjugated anti-rabbit secondary antibody at a 1:1000 dilution was incubated with the membrane for another 1 hour at room temperature. The protein was detected with enhanced chemiluminescence (Pierce, Rockford, IL) for autoradiography (Hyperfilm-Pharmacia).

### Immunoblot and immunoprecipitation assay for Bcl-2 family proteins

To determine the changes in the expression of Bcl-2 family proteins in TM40D cells, cells induced under different procedures were collected and homogenized in lysis buffer and were sonicated for 4 times for 5 seconds each on ice. The lysis mixture was centrifuged at 14,000 rpm for 15 min. Sample protein concentration was measured using the method of Bradford (Bio-rad). Bax protein level was determined using conventional Western blot analysis. For the treatment of cycloheximide (CHX), TM40D-cont and TM40D-Mp (18) cells were first induced with STS for 1 hour before the addition of CHX (50 μM) at the time of 0, 2, 3, and 4 hrs. Vehicle treated tumor cells for 4 hr were used as control. Bcl-2 and Bcl-X_L _protein were determined by immunoprecipitation assay. Briefly, 1,000 μg of protein lysis was incubated with 1 μg of anti-Bcl-2 or anti-Bcl-X_L _antibodies overnight at 4°C, respectively. Twenty microliters of protein A agarose beads (Santa Cruz) were then added to the mixture for an additional 4 hours at 4°C. Immunoprecipitates were harvested by centrifugation at 2,500 rpm for 5 min at 4°C. The beads were washed 5 times in lysis buffer. Pellets were resuspended in 20 μl of 3× SDS samples buffer and heated for gel electrophoresis.

For Western blot analysis of Bcl-2 family proteins, samples were run in 15% SDS polyacrylamide gels. The proteins were transferred onto a PVDF membrane (Bio-rad) and blotted with anti-Bax (1:1000), anti-Bcl-2 (1:1000) and anti-Bcl-X_L _(1:500). Horseradish peroxidase-conjugated anti-rabbit secondary antibody at a 1:1000 dilution was incubated with the membrane for another 1 hour at room temperature. The protein was detected by enhanced chemiluminescence (Pierce, Rockford, IL) and autoradiography (Hyperfilm-Pharmacia).

### Ribonuclease protection assay

For ribonuclease protection assays (RPA), total RNA was isolated from TM40D-cont and TM40D-Mp cells treated by staurosporine (1 μM) for 4 hrs. ^35^S labeled Bcl-2 family antisense probes were generated by transcription of Bcl-2 family genes using a RPA kit (BD PharMingen, Inc., CA) in accordance with the manufacturer's instruction. Protected bands were exposed to an X-ray film and quantified by phosphoimaging analysis. Relative RNA levels were obtained by dividing the quantitated volume of the protected Bcl-2 family gene bands by the volume of either GPADH or L32 control.

### Statistical analysis

The differences of means between groups were assessed by the Student's *t*-test. P < 0.05 was considered significant (two tail analysis). All statistical analysis was performed using Microsoft office XP Software.

## Results

**Figure 1:**
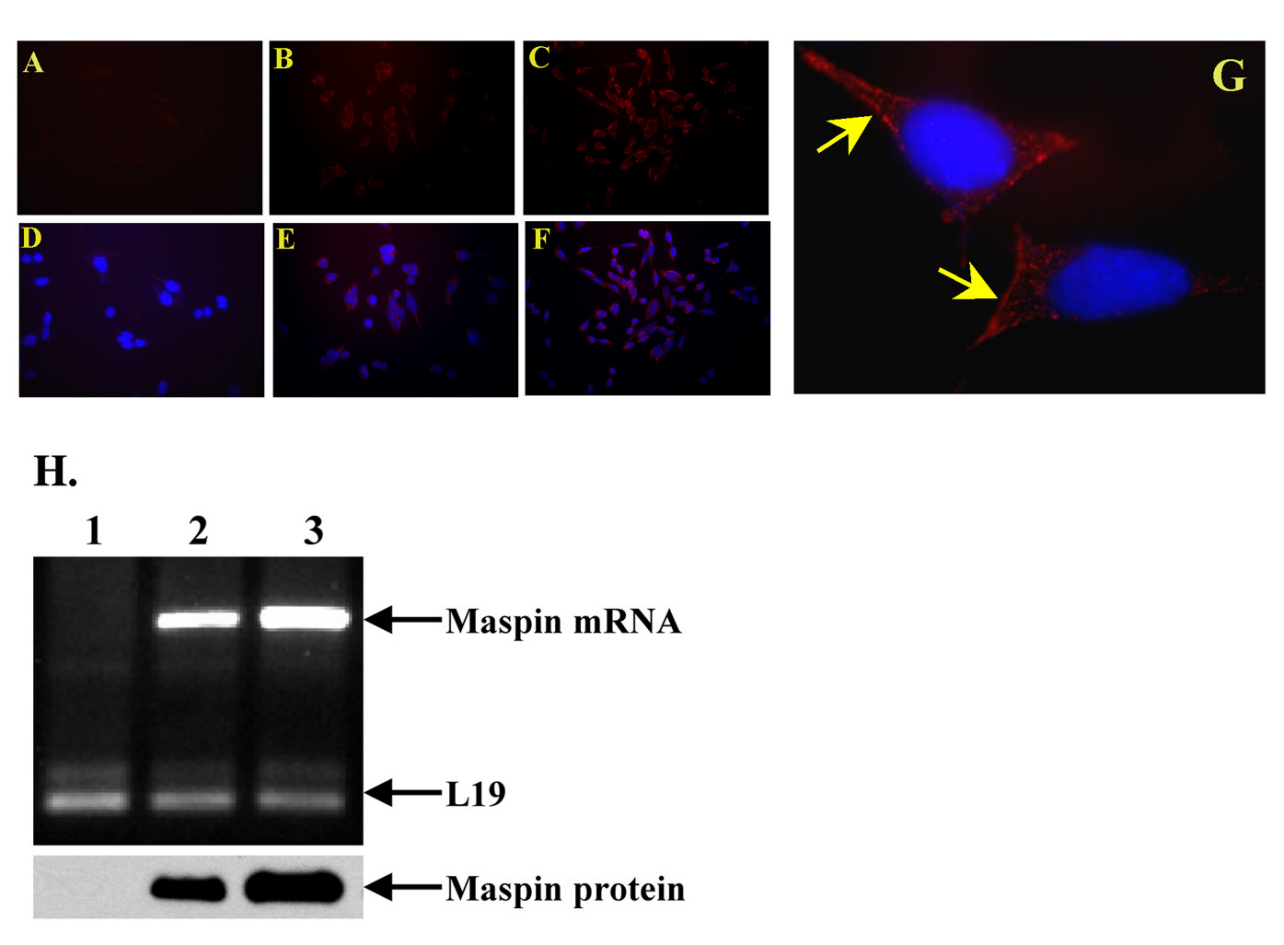
Maspin expression in TM40D stable cell lines. Cells were stained with an AbS4A maspin primary antibody and followed by Texas-red conjugated secondary antibody. Immunofluorescence staining was carried out using TC (A, C), T16 (B, E), and T18 cells (C, F). G. High magnification of immunofluorescence staining of T18 (100× oil len) under non-permeabilized condition. Arrows indicate cell surface staining of maspin. Nuclei were stained with DAPI (D, E, F, and G). H. Quantitation of maspin expression in TC (lane 1), T16 (lane 2), and T18 (lane 3) by RT-PCR and Western blot analysis. L19 serves as a loading control for RT-PCR analysis.

To examine whether maspin is involved in the induction of tumor cell apoptosis, stable clones with various levels of maspin expression were selected and analyzed using semi-quantitative RT-PCR, immunostaining, and Western blot analyses. As shown in Fig. 1, two maspin expression clones TM40D-Mp (16) (T16) and TM40D-Mp (18) (T18), and a negative control subclone TM40D-cont (TC) were selected for further study. We examined the localization of maspin in maspin expressing and control tumor cells using immunofluorescence staining with an anti-maspin antibody. T16 and T18 had detectable immunostaining signals (B, C, E, and F) while TC did not express maspin (A, D). Under high magnification, we observed that maspin immunostaining was abundantly located in the cytoplasm and on the cell surface (Fig. 1G). T18 cells had a higher level of maspin expression than T16 cells at both mRNA and protein levels (Fig. 1H).

**Figure 2:**
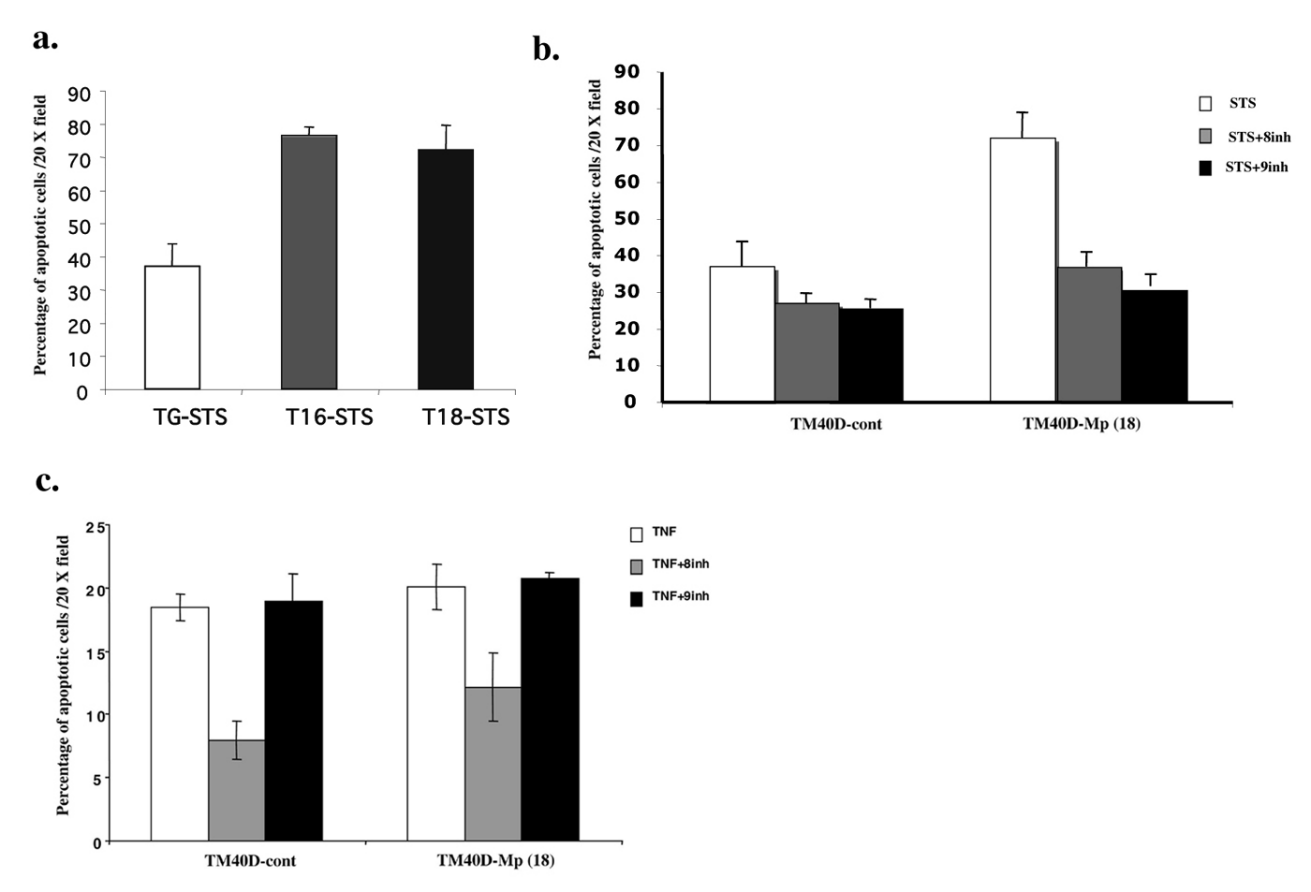
Detection of an increased apoptosis in maspin expressing tumor cells in cell culture under the induction of STS and TNF-alpha. A. TC, T16, and T18 cells in monolayer culture were treated with staurosporine (1 μM) for 4 hrs, and apoptosis rate was detected using the TUNEL assay. Note the increased apoptosis in T16 and T18 cells. B, C, Maspin-mediated apoptosis was blocked by caspase 9 and caspase 8 inhibitors. B. TC and T18 cells were treated with staurosporine (1 μM, 4 hrs), caspase-8 inhibitor II (Z-IETD-FMK) and caspase-9 inhibitor II (LEHD-CHO) were used at a concentration of 20 nM. C. TC and T18 cells were treated with TNF-alpha (100 ng/ml, 7 hrs), caspase-8 inhibitor II (Z-IETD-FMK) and caspase-9 inhibitor II (LEHD-CHO) were used at a concentration of 20 nM. Apoptotic cells were assayed by TUNEL and counted in four randomly selected fields under 20 X objective. Bars were from four repeated experiments.

TC, T16, and T18 cells were treated with chemical reagent staurosporine (STS) or TNFα. As shown Fig. 2A, TC, T16, and T18 cells underwent different levels of apoptosis when they were treated with STS. Caspase 8 and caspase 9 inhibitors were added to TC and T18 cells treated with STS and TNFα [16,17]. In both TC and T18 cells, the rate of apoptosis in STS-treated cells was inhibited when cells were treated with caspase 9 and 8 inhibitors, more so in T18 cells than that in TC cells (Fig. 2B). In contrast, when TC and T18 cells were induced to apoptosis with TNF-α, only caspase 8 inhibitor inhibited tumor cell apoptosis (Fig. 2C). However, there was no difference in the rate of apoptosis between T18 and control TC cells when they were induced with TNF-α (Fig. 2C).

**Figure 3:**
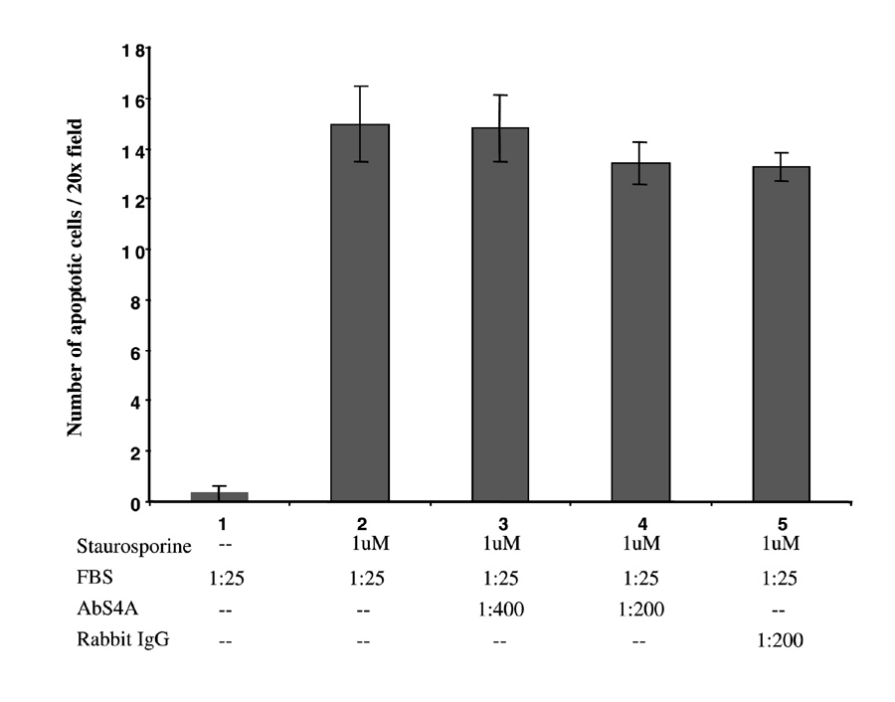
Maspin mediated apoptosis is not through a cell surface event. T18 cells in monolayer culture were treated with staurosporine (1 μM). Maspin blocking antibody AbS4A at two concentrations (from a dilution of 1:200 to 1:400) was added to the cell culture. After 7 hours incubation, cells were fixed for TUNEL staining and apoptotic cells were counted in four randomly selected fields under 20 X objective. Results were from three repeated experiments.

Serpins can regulate cellular apoptosis through both extrinsic and intrinsic pathways [18,19]. If maspin is involved in an extrinsic pathway, it will require that the death signal be initiated by extracellular maspin protein. Since maspin is present in both cytoplasm and on the cell surface in TM40D-Mp cells (Fig. 1D), it is possible that maspin could be secreted and bound to the surface of tumor cells. One major function of maspin is the inhibition of tumor cell migration which is mediated through a cell surface event [20]. One maspin antibody Abs4A acts as function-blocking reagent in the migration assay [20]. To further determine whether maspin-mediated apoptosis is through secreted maspin protein, an anti-maspin AbS4A polyclonal antibody was incubated with T18 cells that were stimulated for apoptosis with staurosporine. As shown in Fig 3, AbS4A maspin antibody did not block STS-induced cell apoptosis at concentrations up to 1 μg/ml (1:200 dilution), even though the same antibody was effective at blocking maspin's effects on cell migration at a much lower dosage (data not shown). This suggests that secreted maspin from TM40D-Mp cells does not induce cell apoptosis through secreted maspin acting on the cell surface.

**Figure 4:**
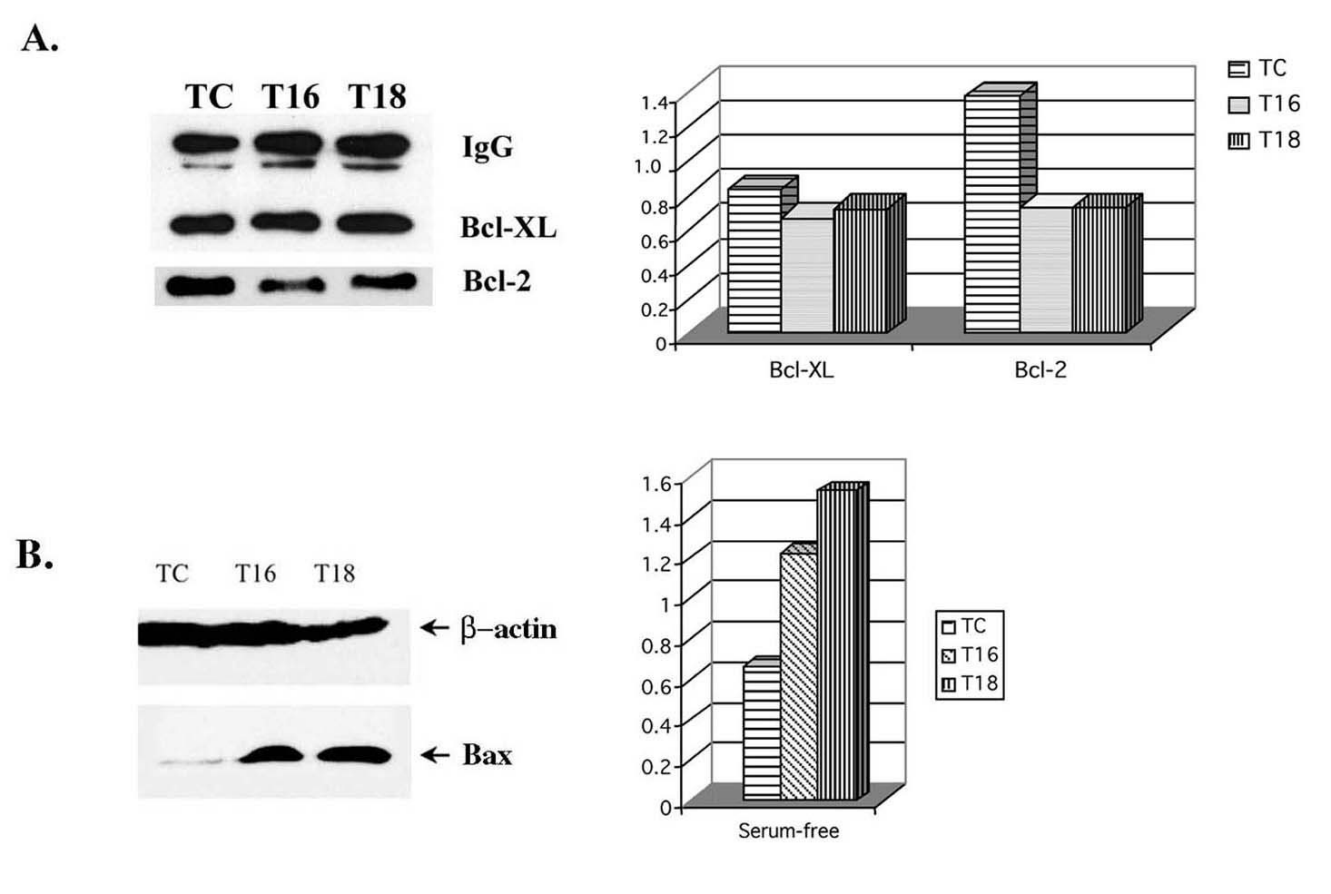
Changed levels of Bcl-2 family proteins in TC, T16, and T18 tumor cells. A. Analysis of anti-apoptotic Bcl-2 proteins in tumor cells. Cells were cultured for 48 hrs in serum starvation condition before they were lysed in cell lysis buffer. Cytosolic proteins (1 mg) were immunoprecipitated with Bcl-2 and Bcl-X_L _monoclonal antibodies overnight at 4°C. The level of Bcl-2 and Bcl-X_L _proteins was detected using a HRP-conjugated anti-mouse secondary antibody. IgG serves as an internal control. Right panel, relative level of Bcl-2 and Bcl-X_L _proteins in TC, T16, and T18 cells. The protein bands were quantitated using a densitometer and were normalized to IgG signals. B. Analysis of pro-apoptotic Bax protein in TM40D cells. Cells were induced to apoptosis with serum starvation. Cell extracts were run on SDS PAGE gel and immunobloted with anti-Bax and anti-beta-actin antibodies. Beta-actin serves as a loading control. Right panel, relative level of Bax protein in TC, T16, and T18 cells. The protein bands were quantitated using a densitometer and were normalized to beta-actin signal.

The Bcl-2 family proteins consist of pro- and anti- apoptotic proteins that act through mitochondria to regulate apoptosis through the caspase 9 and cytochrome c pathway. Since we know that maspin-mediated apoptosis involves mitochondrial death pathway [13], we examined the protein level of three key apoptosis proteins in tumor cells treated under serum starvation. These proteins include Bax, a pro-apoptotic protein [21], and the anti-apoptotic Bcl-2 and Bcl-X_L _[21-23]. When these cells were serum starved to induce apoptosis, the levels of anti-apoptotic proteins such as Bcl-2 and Bcl-XL were too low to be detected by conventional Western blot analysis. Thus, we used the method of immunoprecipitation followed by Western blot analysis to analyze their expression levels. No difference was detected in Bcl-XL protein between the maspin expressing T16 and T18 cells, and the control TC cells (Fig. 4A). However, a decrease was consistently observed in Bcl-2 level in T16 and T18 cells compared to TC cells under serum starvation condition (Fig. 4A). The pro-apoptotic protein Bax could be easily detected when tumor cells were placed under serum starvation condition. In contrast to Bcl-2 protein, a dramatic increase in Bax protein level was observed in T16 and T18 cells compared to TC cells (Fig. 4B). Such increased level of Bax was not observed when cells were treated with TNF-alpha, which mediates cell apoptosis through death receptor rather than the mitochondrial pathway (data not shown).

**Figure 5:**
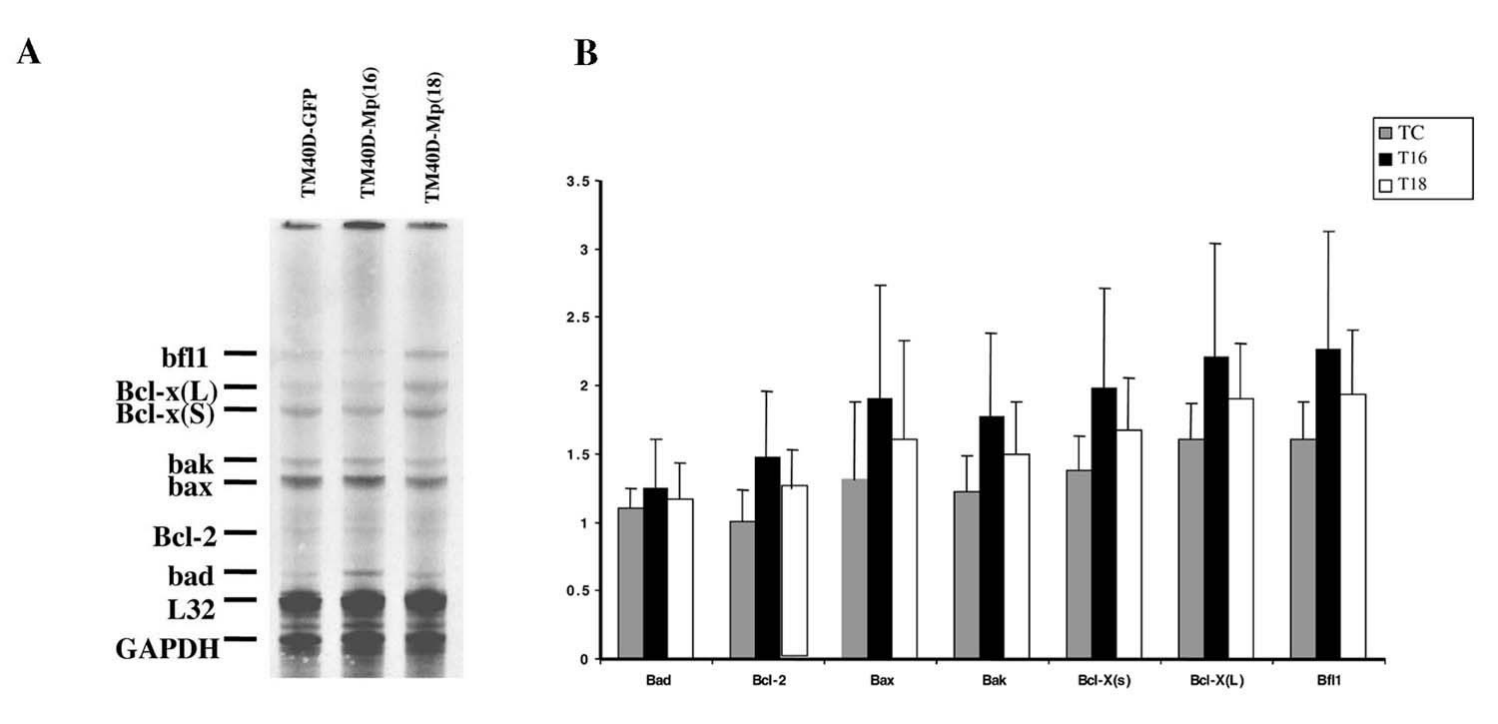
Expression profile of Bcl-2 family genes in TC, T16, and T18 tumor cells. A. Ribonuclease protection assay (RPA) for Bcl-2 family genes. Antisense radiolabeled multiprobes were prepared using a RPA kit for detection of Bcl-2 family genes. B. Relative level of Bcl-2 family genes quantitated using a densitometer. Results are from three RPA experiments. Error bars represent standard deviation. No significant difference (p >> 0.05, t-test) in the expression of any Bcl-2 family genes was observed for TM40D-cont (TC), and TM40D-Mp (T16, T18) cells.

To determine whether maspin overexpression regulates the gene expression of Bcl-2 family during apoptosis, a quantitative ribonuclease protection assay (RPA) was carried out using radiolabeled antisense probes for Bcl-2 family genes (RPA kit, BD PharMingen, Inc., CA), and RNAs isolated from tumor cells under apoptotic condition. As shown in Fig. 5, the levels of Bax, Bcl-2, and several other Bcl-2 family genes were not changed significantly between control TC cells and maspin expressing T16 and T18 cells, suggesting the regulation is not at the transcription level.

**Figure 6:**
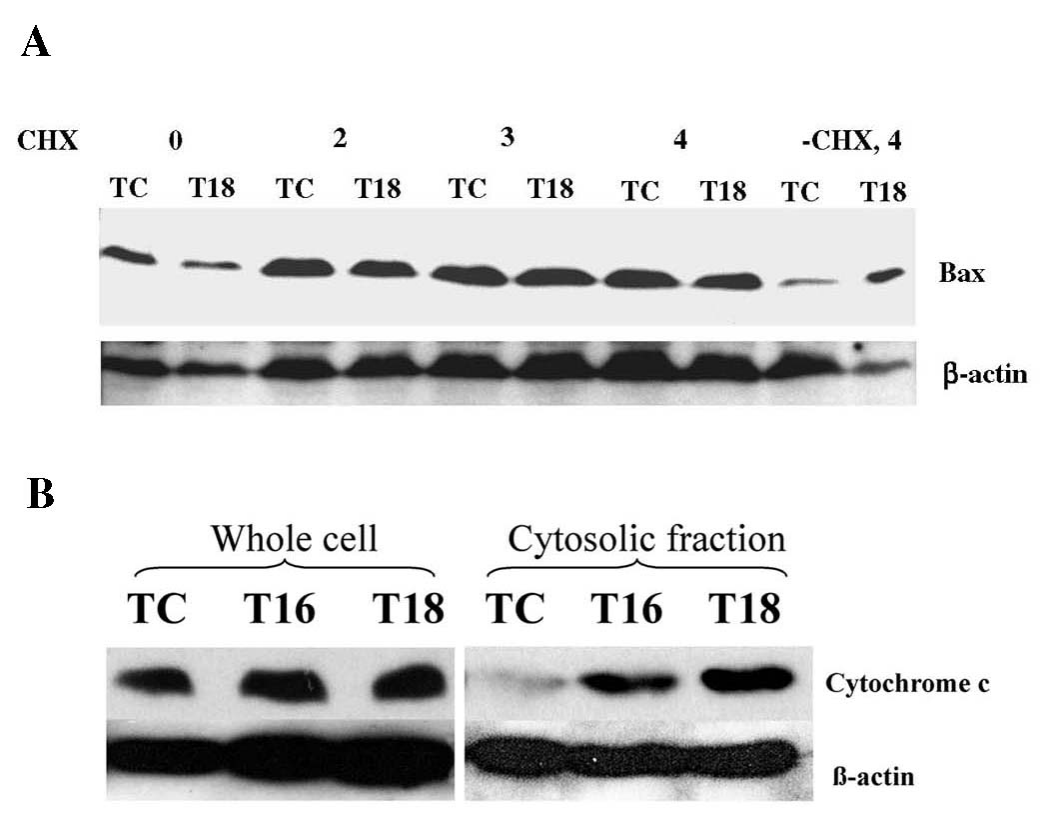
Maspin regulates the stability of Bcl-2 family proteins and the release of cytochrome c from mitochondria. A. Bax stability in TM40D cells. TC and T18 cells were induced with STS (1 μM) for 1 hour before they were treated by CHX (50 μM) for 0, 2, 3, and 4 hours. DMSO was used as a vehicle control (-CHX, 4 hrs). β-actin was used as a loading control. B. Release of cytochrome c protein from mitochondria to cytosol. TC, T16, and T18 tumor cells were treated with serum starvation for 48 hrs before they were harvested for preparation of cell extract and for cellular fractionation. Mitochondria were removed from cytosolic fraction by centrifugation. Both whole cell extracts and cytosolic fractions were subjected to Western blot analysis using antibodies against cytochrome c and beta-actin. β-actin was used as a loading control.

Bcl-2 proteins could be controlled at the level of protein stability. The decreased level of Bcl-2 in T16 and T18 cells (Fig. 4) is in line with a previous report demonstrating Bcl-2 protein was specifically targeted for degradation during apoptosis by an ubiquitin-dependent event [24]. Since T16 and T18 cells have higher level of apoptosis, more Bcl-2 proteins could be targeted for degradation in proteasome complex. The Bax level was higher in T16 and T18 cells compared to TC cells (Fig. 4). To determine whether the increased level of Bax protein in T16 and T18 cells is due to increased protein stability, cells were induced to apoptosis with STS along with cycloheximide (CHX), which prevents new protein synthesis. The level of Bax protein in TC cells was as stable as that in T18 cells after CHX treatment for 2, 3, and 4 hrs (Fig. 6A). However, Bax level was specifically reduced in TC cells untreated with CHX compared to TC cells that were treated with both STS and CHX for the same time period (4 hrs). Less Bax protein was degraded in vehicle treated T18 cells than that in vehicle treated TC cells.

A change in the level of Bcl-2 family proteins in TC and T18 cells may increase the sensitivity of cells to apoptotic signal through their downstream components of mitochondrial death pathway. Cytochrome c is a critical component of the cascade. In normal condition, cytochrome c is located in the intermembrane space of mitochondria in the cell, where it functions as a transducer of electrons in the respiratory chain. When cells undergo apoptosis, cytochrome c is released into the cytoplasm, which initiates the mitochondrial death cascade [25]. To further determine whether maspin regulates the cytochrome c release from the mitochondria, TM40D-cont and TM40D-Mp cells were serum starved and the cells were harvested for both whole cell extracts and cellular fractionations without mitochondria. Both the whole cell and cytosolic fractions were collected for Western blot analysis using an anti-cytochrome c antibody. As shown in Fig. 6B, the total amount of cytochrome c in the whole cell extracts of TC and T18 cells remained at similar level. However, maspin expressing T18 cells had an increased release of cytochrome c to the cytosolic fraction compared to the TC cells. This suggests that maspin-mediated apoptosis is through the caspase 9 and cytochrome c death pathway.

## Discussion

We have shown in the past few years that maspin acts as a tumor suppressor, inhibiting both primary tumor growth and metastasis in cell culture and animal models. Here, we provide evidence that maspin is actively involved in the induction of tumor cell apoptosis.

There are at least two general apoptosis pathways, the intrinsic and extrinsic pathway. Although maspin protein is present in cytosol and on the cell surface, the induction of apoptosis is not mediated by extracellular maspin. Treatment of maspin expressing T18 cells with STS and an anti-maspin antibody did not affect the apoptosis rate (Fig. 3). This data is in line with a previous report by Jiang et al. which showed that exogenous maspin failed to sensitize human breast tumors to chemical induced apoptosis [11]. This implicates that intracellular maspin is actively affecting cell apoptosis, which is supported by our report of translocation of intracellular maspin to the mitochondria [13]. Our data also confirmed that in maspin expressing T16 and T18 cells, the Bcl-2 level was decreased and the level of pro-apoptotic protein Bax was significantly increased compared to control TC cells. Such regulation was selectively controlled since another Bcl-2 family protein Bcl-Xl was not affected (Fig. 4A). The effect of maspin on cell apoptotic machinery was not preexisting since both TC and T18 cells had similar, low level of apoptosis in high serum medium. Second, the levels of Bax and caspase 3 were similar before the cells were treated under serum starvation or with STS (data not shown). However, their levels were changed under stress treatment, indicating that the change is dependent on cell death stimulation. The regulation of Bcl-2 genes by maspin was not at the transcriptional level (Fig. 5A). Rather, the stability of these proteins was affected by maspin. Others have shown that Bcl-2 protein was dephosphorylated and targeted to ubiquitin-dependent degradation during apoptosis in HUVEC cells [24]. Phosphorylation of Bcl-2 confers resistance against induction of apoptosis. Since Bcl-2 controls the activation of caspase cascade by its interaction with Bax and by participation in apoptosome ensemble, degradation of Bcl-2 may unleash the inhibitory function of Bcl-2 over the apoptosome [26]. The unexpected finding is that the pro-apoptotic Bax was stabilized in the presence of maspin through an unknown mechanism. When new protein synthesis was inhibited by cycloheximide, including those proteins control protein degradation, Bax protein was as stable in TC cells as in maspin expressing T18 cells. Such event of ordered degradation of proteins happens for the regulation of p53 protein during the cell cycle control [27,28]. It is possible that when T16 and T18 cells were induced for apoptosis, more Bax protein might oligomerize and translocate to mitochondria [29,30], which might make the protein less susceptible for degradation in the cytosol. However, in control TC cells treated under apoptotic stress, less Bax proteins might oligomerize and move to mitochondria and thus were more sensitive to degradation in the cytosol. A recent study from my laboratory showed that during maspin-mediated apoptosis, there was also an increased translocation of maspin from the cytoplasm to the mitochondria [13]. Whether there is any connection between Bax and maspin translocation during apoptosis remains to be tested.

The finding that maspin-induced apoptosis was partially blocked by both caspase 8 and caspase 9 inhibitor is interesting. Several other reports also show that certain reagents including STS can induce caspase 3 activity and apoptosis through both the activation of caspase 8 and caspase 9 [31-35]. In addition, there are numerous reports demonstrating the cross-talk between caspase 8 and 9 [36,37]. At the moment, no data suggest that maspin-mediated apoptosis is connected to the death receptor pathway. Rather, our previous report and this study demonstrate that at least the mitochondrial mediated apoptosis pathway is involved [13]. Finally, our data also indicate that the increased apoptosis in maspin expressing cells is due to changes in Bcl-2 family protein level. Due to the changed level of Bcl-2 proteins, the apoptotic pathway became more active in maspin-expressing cells and more cytochrome c proteins were released from mitochondria to the cytosol (Fig. 5B). In many cancers, the anti-apoptotic protein Bcl-X_L _and Bcl-2 are found to be over-expressed while the activity of pro-apoptotic Bax is counterbalanced by strong surviving signals [38]. Our finding suggests that one of the functions that maspin plays is to change the level of Bcl-2 family proteins in tumor cells. It is noted that the increased apoptosis mediated by maspin was observed under both serum starvation and chemical drug induction, confirming that the existence of a general mechanism of action through the intracellular, mitochondria death pathway. Such findings offer a new direction for cancer therapy. Identifying the molecular mechanism of maspin-mediated apoptosis will help us to design better reagents for maspin-based therapeutic interventions against breast cancer.

## Conclusion

Overexpression of maspin in mouse mammary tumor cells increased the rate of tumor cell apoptosis when they were treated with STS or serum starvation. Maspin-mediated apoptosis could be partially blocked by caspase 9 and caspase 8 inhibitors. The effect was not through extracellular maspin. Maspin overexpression in breast tumor cells regulated the level of Bcl-2 family proteins. Maspin-expressing T16 and T18 cells had a reduced level of anti-apoptotic protein Bcl-2 but an increased level of pro-apoptotic protein Bax compared to control TC tumor cells. Such regulation occurred at the level of protein stability. The change in Bcl-2 family proteins resulted in an increased release of cytochrome c from mitochondria, thus the increased apoptosis in maspin-expressing tumor cells.

## Competing interests

The author(s) declare that they have no competing interests.

## Authors' contributions

WZ Performed the experiments.

YS Performed the experiments.

MZ Conceived and coordinated the study.

## Pre-publication history

The pre-publication history for this paper can be accessed here:
